# The Brain Toxin Cleansing of Sleep Achieved During Wakefulness

**DOI:** 10.3390/jcm14030926

**Published:** 2025-01-31

**Authors:** Gary W. Arendash

**Affiliations:** RF Longevity, 428 E. Thunderbird Rd., Suite 431, Phoenix, AZ 85022, USA; arendash@rflongevity.com; Tel.: +1-(480)-395-1481

**Keywords:** brain toxin cleansing, wakefulness, radiofrequency waves, Alzheimer’s Disease, meningeal lymphatic vessels

## Abstract

A primary purpose of sleep for humans is to remove toxins and metabolic wastes from the brain (e.g., Aβ, tau, lactate) that would otherwise build up and compromise brain functionality. There are currently no drugs or devices that have been clinically shown in humans to enhance brain toxin removal, either during sleep or wakefulness. This perspective article focuses on a recently (re)discovered major route of toxin drainage from the human brain through meningeal lymphatic vessels (mLVs) and the primary enhancer of their flow—the cytokine Vascular Endothelial Growth Factor (VEGF). The purpose of this perspective article is to present pre-clinical and clinical evidence relevant to a new bioengineered technology (Transcranial Radiofrequency Treatment; TRFT) that appears to enhance mLV flow to increase brain toxin cleansing in humans during wakefulness. In being both safe and non-invasive, TRFT is administered in-home, presently through a device called “MemorEM”. Two months of daily TRFT during wakefulness increased the typically low plasma/brain levels of VEGF in Alzheimer’s Disease (AD) subjects, which was associated with increased Aβ and tau toxin removal from their brains during wakefulness—ostensibly through VEGF-increased mLV flow. Even irrespective of baseline VEGF levels, brain toxin cleansing was increased by TRFT in AD subjects, who also experienced a notable reversal of their cognitive impairment after TRFT. Additional clinical studies are nonetheless required to firmly establish TRFT’s brain cleansing abilities during wakefulness. In performing a major duty of sleep, TRFT during wakefulness is proposed as a viable intervention to counter the decline in nighttime brain toxin cleansing that occurs with aging and in multiple brain diseases, most notably Alzheimer’s Disease. The implications of TRFT for insomnia and for sleep deprivation are also discussed, as is the potential for TRFT to extend healthy human longevity.

## 1. Introduction: Is There an Alternative to Sleep for Cleansing the Human Brain of Toxins and Metabolic Wastes?

A major purpose of sleep is to cleanse the brain of toxins (e.g., Aβ, tau) and metabolic wastes (e.g., lactate, components of degenerating neurons, extracellular vesicles) that would otherwise build up and compromise normal brain function [1,2,3,4]—thus, 8–9 h per day of sleep for adults is recommended to remain alert and healthy during wakefulness. It is well known that adults at an older age often sleep less than the recommended number of hours [5]. In that regard, less sleep and sleep disturbances are strongly linked to the development and greater progression of Alzheimer’s Disease (AD) [6,7,8], with around half of AD subjects having sleep disorders [9].

It is, however, intriguing that some of the greatest minds in history slept much less than the aforementioned recommendation of 8–9 h per day. Nichola Tesla and Leonardo Davinci slept only 2–3 h per night, while Thomas Edison slept no more than 3–4 h in any given 24 h period [10,11,12]. All three of these men believed that sleep was counter to their productivity and something that should be minimized to the extent possible. So, is it possible that these great minds had a more effective way of cleansing toxins/metabolic wastes from their brains than just during sleep? Can the toxin cleansing and metabolic waste removal performed by our brains be made to occur, at least in part, during wakefulness to then reduce the number of sleep hours needed daily to maintain normal brain function and good health?

This perspective article discusses the clinical administration of safe, non-invasive radiofrequency (RF) waves to the entire human forebrain via a medical device (MemorEM) and how it increases the drainage/cleansing of brain toxins during wakefulness—ostensibly through enhancement of the brain’s meningeal lymphatic flow. The published clinical studies with TRFT, particularly the most recent clinical findings [13,14], appear to be the first to provide soluble biomarker evidence of increased brain cleansing by any human clinical intervention. It is proposed that Transcranial Radiofrequency Wave Treatment (TRFT) during wakefulness can “rejuvenate” the brain cleansing that is reduced during aging and in multiple brain diseases of aging (e.g., Alzheimer’s Disease, Parkinson’s Disease, Stroke, and Multiple Sclerosis) [15,16,17,18,19]. Also discussed is the potential for TRFT to counter the deleterious effects of insomnia/sleep deprivation on brain toxin cleansing/metabolic waste removal, and for TRFT to even reduce the amount of sleep normally required for brain toxin cleansing.

In 2020, both the MemorEM device and Transcranial Electromagnetic Field Treatment (TEMT) technology were given “breakthrough” designation by the FDA against AD memory decline in recognition of TEMT’s neurotherapeutic potential. However, in order to distinguish our use of electromagnetic waves in the RF range from other neuromodulatory approaches using much higher electromagnetic wave frequencies (e.g., infrared and light), we prefer the term TRFT for describing our neuromodulatory approach utilizing RF waves. As such, although the term “TEMT” was utilized in all of our pre-clinical publications through 2016 and clinical publications through 2023, the term “TRFT” was utilized in our most recent 2024 and 2025 publications, as well as in this current perspective article. The technology and the MemorEM device are identical for both designations.

## 2. Meningeal Lymphatic Vessels: A Major Route for Drainage of Human Brain Toxins

**Figure 1:**
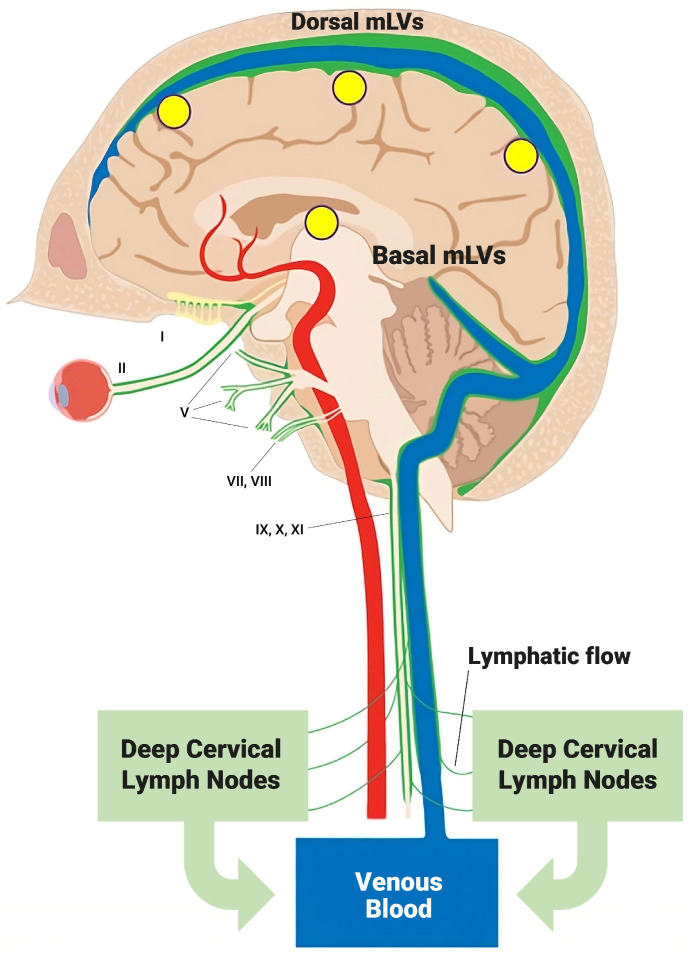
Meningeal lymphatic vessels (mLVs), located within the brain’s meninges/dura, consist of both “Dorsal” and “Basal” lymphatic components (green vessels). Collectively, these lymphatic vessels account for up to half of total brain cerebrospinal fluid (CSF) drainage out of the brain, resulting in a substantial amount of toxin drainage/clearance from the brain. CSF within mLVs is first transported to cervical lymph nodes and then into the venous circulation. Note the close parallel relationship of mLVs to venous sinuses (blue) within the brain. Arterial blood supply to brain is in red. Yellow circles depict the approximate head surface locations on the left side of the head for the four radiofrequency emitters of a MemorEM device. Figure adapted from Ref. [14].

There are two primary routes through which toxins/metabolic wastes within cerebrospinal fluid (CSF) leave the human brain to enter venous blood for their metabolism/body removal. First, CSF can flow directly into the brain’s venous sinuses via arachnoid villi. Second, CSF can flow into the brain’s dorsal and ventral meningeal lymphatic vessels (mLVs), located adjacent and parallel to the brain’s venous sinuses, then to cervical lymph nodes, and finally into venous blood (Figure 1).

Upstream from both of these routes is the “glymphatic system”, which provides toxin clearance from the brain’s parenchyma itself through bulk CSF/interstitial fluid flow that occurs near-exclusively during sleep [20]. Although some form of parenchymal toxin clearance is likely, issues and inconsistencies involving the originally described glymphatic system have been recently raised [21]. Irrespective of what form brain “parenchymal” toxin clearance takes, it is tangential to this paper’s focus on the down-stream targeting of CSF/brain toxin drainage via mLVs.

**Figure 2:**
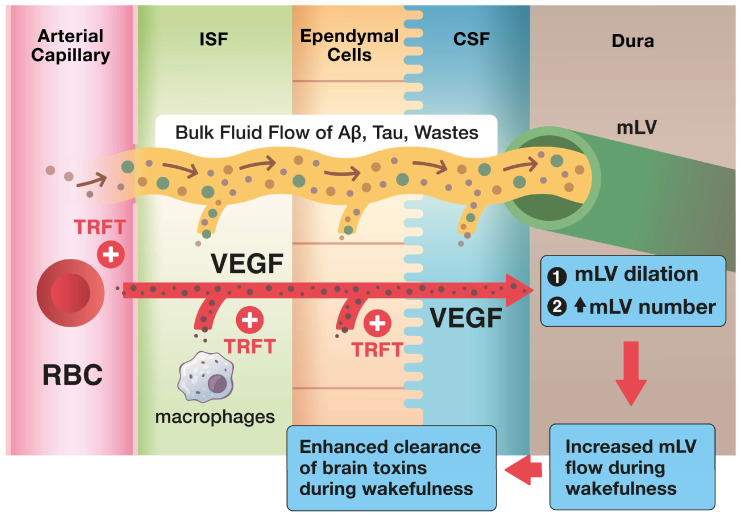
Extensive toxin (e.g., Aβ, tau) removal/clearance from the human brain takes place through increased mLV flow of CSF out of the brain. This occurs with dilation of mLVs and/or an increase in their numbers (lymphangiogenesis). As a critical cytokine that enhances both of these processes, Vascular Endothelial Growth Factor (VEGF) increases CSF flow and toxin removal from the brain. The three likely sources of VEGF that modulate mLV diameter and vessel numbers are (1) VEGF in blood plasma of choroid plexus capillaries that diffuse to mLVs, (2) ependymal cells lining the choroid plexus, and (3) resident macrophages within the choroid plexus interstitial fluid; both 2 and 3 also result in VEGF diffusion to mLVs. Transcranial Radiofrequency Wave Treatment (TRFT) likely affects all three of these VEGF sources [14].

Of the two aforementioned routes for CSF/toxin cleansing from the brain, the mLV route is responsible for up to half of total brain CSF/toxin drainage (Figure 1 and Figure 2) [22,23]. The importance of mLVs for toxin drainage from the brain is underscored by studies showing that the blockage of mLV drainage in AD transgenic mice increases their brains’ accumulation of the toxin β-amyloid (Aβ) [24] and that transgenic mice lacking a functional mLV system have higher t-tau retention in their brains [25]. Importantly, mLV flow/activity occurs primarily during sleep and is very markedly reduced during wakefulness [1,26]. Accordingly, there is a 95% reduction in Aβ removal from the brain during wakefulness compared to during non-REM sleep, when mLV flow/activation is at its greatest [1]. Multiple studies have reported that mLV drainage decreases with aging and various age-related diseases/disorders (e.g., Alzheimer’s Disease, Parkinson’s Disease, Stroke, multiple sclerosis) [19]. Thus, an intervention that can rejuvenate mLV drainage in normal-aged individuals, or in those with age-related diseases linked to mLV dysfunction, could be of immense value in re-establishing the balance between brain toxin production and removal.

**Figure 3:**
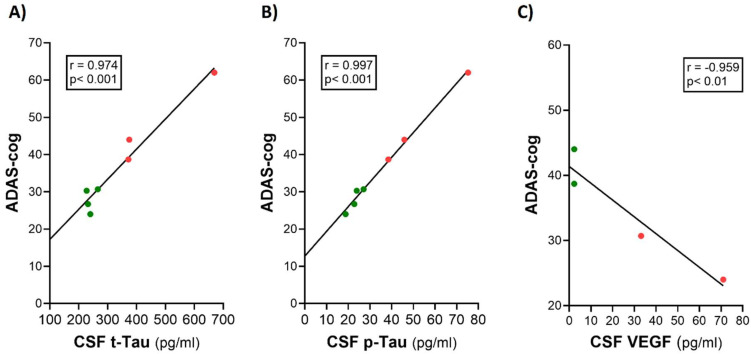
ADAS-cog scores strongly correlate with CSF levels of t-tau (**A**), p-tau (**B**), and VEGF in AD subjects (**C**). Higher levels of t-tau and p-tau were correlated with poorer ADAS-cog performance, while higher levels of VEGF were correlated with better ADAS-cog performance. Red dots represent AD subjects with higher (poorer) ADAS-cog scores, while green dots represent AD subjects with lower (better) ADAS-cog scores. Graphs reproduced from Ref. [14].

As the master regulator of brain mLV drainage, the cytokine Vascular Endothelial Growth Factor (VEGF) induces an increase in CSF flow through mLVs by causing their dilation and also inducing the formation of new mLVs through lymphangiogenesis (Figure 2). There are three main sources of VEGF that can diffuse to and influence mLV flow/function: (1) diffusion of VEGF from plasma within cerebrovascular capillaries, (2) synthesis within ependymal cells lining the choroid plexus, and (3) secretion by resident macrophages within the choroid plexus (Figure 2) [17]. Clinical evidence for VEGF being critical for toxin clearance from the brain and better cognitive function can be appreciated from the presence of strong correlations between cognitive function (ADAS-cog performance) and CSF levels of VEGF, t-tau, and p-tau in AD subjects (Figure 3). Poorer cognitive performance and higher t-tau and p-tau levels are seen in AD subjects with low VEGF levels, while better cognitive performance and lower t-tau and p-tau levels occur in AD subjects with high VEGF levels (Figure 3). It is proposed that these relationships in AD subjects involve VEGF’s direct ability to increase mLV flow, resulting in the enhanced brain clearance of toxins and better cognitive performance. Supportive of this premise, AD subjects typically have lower serum VEGF levels compared to aged controls [27]. Moreover, the measurement of VEGF in CSF of normal-aged, mild cognitive impairment (MCI), and AD subjects showed that higher VEGF levels (irrespective of group) were strongly correlated with less memory decline [28].

## 3. Increasing mLV Flow in Mice to Enhance Toxin Drainage from Their Brains

**Figure 4:**
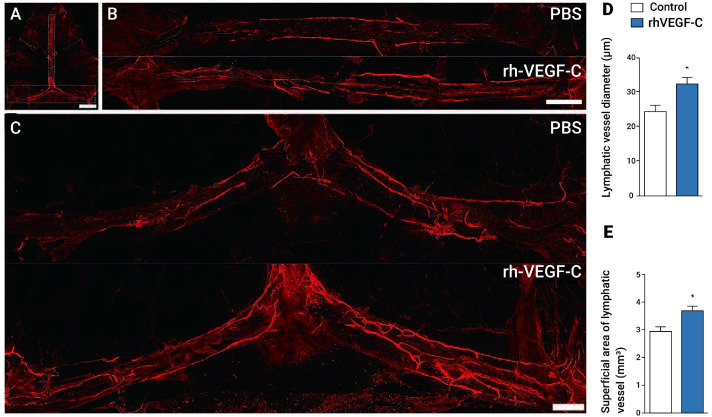
mLV dilation and enhanced lymphangiogenesis in transgenic mice injected with VEGF. In AD transgenic mice, dorsal mLVs are visualized with Lyve-1 and Prox1 immuno-labeling of the meninges after either recombinant VEGF (rh-VEGF-C) or control/PBS intracerebral injections every other day over eight days. (**A**) Location of mLVs analyzed along the superior sagittal sinus (SSS) and transverse sinus (TS). (**B**,**C**) Increased diameter and lymphangiogenesis of mLVs after intracerebral VEGF treatments. In quantification, the mean diameter of dorsal mLVs (**D**) and the total superficial area of dorsal mLVs (**E**) were significantly increased in the VEGF group compared with controls. Quantitative data presented as mean ± SEM. Scale bars: 1000 μm (**A**), 500 μm (**B**,**C**). * *p* < 0.05. Figures/graphs reproduced from Ref. [29].

Increasing mLV flow through VEGF or possibly similar therapeutics would seem to be an ideal way to clear toxins from the human brain, both during normal aging and in age-related diseases. However, prior to the presently described bioengineered technology, no human studies to enhance mLV flow had been conducted. Only animal studies have been conducted, which showed the ability to increase mLV flow primarily through transcranial delivery/injections of VEGF directly into the brains of mice. For example, the transcranial delivery of VEGF in aged mice increased the diameter of mLVs, increased mLV drainage of a CSF tracer into cervical lymph nodes, and improved cognitive performance [15]. In a study involving AD transgenic mice [29], intracranial injections of VEGF induced an increased diameter and number of dorsal meningeal lymphatic vessels (lymphangiogenesis) (Figure 4). These VEGF effects led to the increased clearance of soluble Aβ from the brains of these AD aged mice, as evidenced by higher levels of soluble A-beta1-40 and 1-42 in cervical lymph nodes, as well as improved cognitive performance [29]. Thus, it seems clear that introducing VEGF into the brains of aged or AD transgenic mice can target mLVs to increase their flow, thus increasing toxin clearance from the brain and improving cognitive performance.

Unfortunately, the aforementioned transcranial/intracranial injections of VEGF to increase mLV flow are invasive and impractical for humans, especially for long-term treatment. So, might there be a non-invasive way to increase brain VEGF levels in humans to enhance mLV flow during wakefulness and thus increase brain toxin cleansing during wakefulness? The present paper discusses recent clinical studies that would seem to answer that question with a resolute “yes”.

## 4. Transcranial Radiofrequency Wave Treatment During Wakefulness for Human Brain Toxin Cleansing

**Figure 5:**
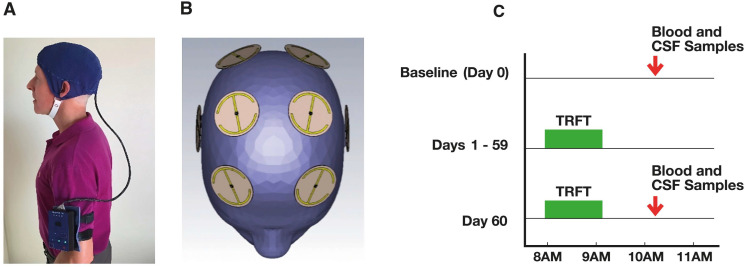
(**A**) A MemorEM device, which provides full forebrain TRFT, is being worn by an individual. Worn on the upper arm is the control panel/battery box, which is wired via a cable to eight radiofrequency wave emitters in the head cap. (**B**) Location of the eight radiofrequency (RF) wave emitters within the head cap, which collectively provide RF treatment to the entire human forebrain. Locations of the four RF emitters on one side of the human head are depicted in Figure 1. (**C**) TRFT occurred in the morning daily during wakefulness for 2 months, with blood and CSF samples taken at baseline and following the final (Day 60) TRFT. Figures (**A**,**B**) are reproduced from Ref. [33].

Multiple recent clinical studies are supportive of TRFT increasing brain toxin cleansing during wakefulness [13,14,30,31]. In a single-arm treatment study [14], baseline venous blood and CSF samples (via spinal tap) were taken clinically from eight mild/moderate AD subjects prior to the start of in-home 1 h TRFT treatments with MemorEM devices (Figure 5A) as manufactured by NeuroEM Therapeutics, Inc. (Tampa, FL, USA) and administered by caregivers. Embedded within each MemorEM device head cap are eight RF wave emitters (Figure 5B) that collectively provide full forebrain RF treatment to the human forebrain due to resultant electric fields penetrating significantly to the forebrain’s center [32,33]. Each of the eight emitters are activated in a sequential fashion at 217 Hz, with any given emitter providing RF treatment at a 915 MHz frequency and 1.6 W/kg power level (Specific Absorption Rate) when in the “ON” position [14].

Following baseline blood and CSF samples, two in-home treatments were then given daily for two months—a morning treatment starting around 8:00 AM and a late afternoon treatment starting around 4:00 PM. On the final day of treatment, blood and CSF were taken clinically within several hours of the morning treatment (Figure 5C). Thus, TRFT was always administered during wakefulness and blood/CSF samples were always taken in the late morning within several hours of completing the morning 1 h TRFT.

**Figure 6:**
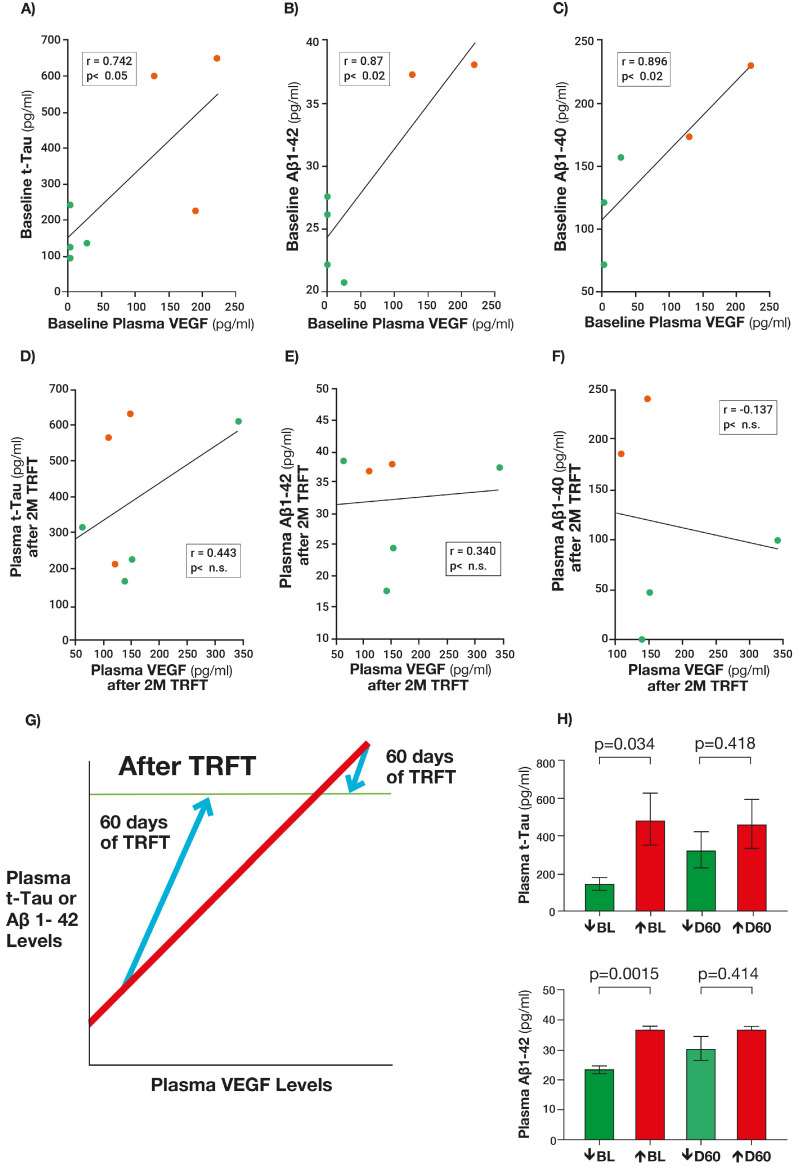
Plasma t-tau, Aβ1-40, and Aβ1-42 levels are directly correlated with plasma VEGF levels (**A**–**C**). Two months of TRFT during wakefulness re-balanced these AD markers to eliminate their correlations with VEGF (**D**–**F**). Strong TRFT-induced increases in plasma VEGF and AD markers were seen in subjects who had low baseline plasma VEGF levels, while small or no TRFT-induced decreases occurred for those AD subjects who had high baseline VEGF levels. Subjects with low baseline (BL) levels of VEGF are indicated by green circles, while those with high BL VEGF levels are indicated by red circles. (**G**) A summary graph showing that this re-balancing of AD markers by TRFT shown in (**D**–**F**) primarily involved an increase in AD marker levels in subjects with low BL levels of VEGF to eliminate correlations. (**H**) The re-balancing of plasma t-tau and Aβ1-42 levels by TRFT is further evident by the significant differences between low versus high baseline (BL) VEGF groups and their elimination at D60 (2 months of TRFT). All graphs, except for (**G**), are reproduced from Ref. [14].

At baseline, three AD markers in plasma (t-tau, Aβ1-42, and Aβ1-40) were directly correlated with plasma VEGF levels (Figure 6A–C), consistent with higher levels of plasma VEGF resulting in increased clearance of all three AD markers from brain/CSF into plasma [14]. Daily TRFT for 2 months during wakefulness eliminated these correlations by (1) robustly increasing plasma VEGF and (ensuing) AD markers in those AD subjects with low VEGF baseline levels and (2) inducing small or no decreases in these measures for those AD subjects with high baseline VEGF levels (Figure 6D–F). This “re-balancing” effect of TRFT on toxins in plasma is primarily due to an increase in AD marker levels in subjects with low baseline levels of VEGF in plasma, as depicted in Figure 6G. If AD subjects are divided into two groups (low and high baseline VEGF levels in plasma), the re-balancing of plasma t-tau and Aβ1-42 levels by TRFT is again evident by the significant differences between low versus high baseline VEGF groups for t-tau and Aβ1-42 being eliminated by 2 months of TRFT (Figure 6H).

**Figure 7:**
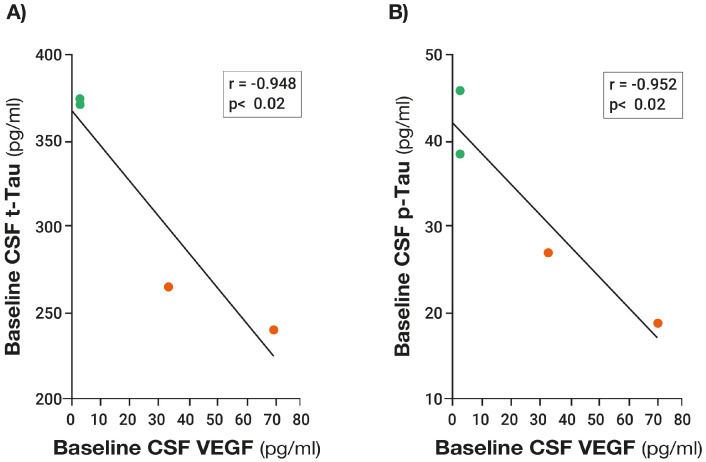
Robust negative correlations were present in CSF between baseline levels of VEGF and baseline levels of both t-tau (**A**) and p-tau (**B**), suggesting that higher VEGF levels were resulting in increased clearance of these AD markers from brain/CSF. Both correlations were eliminated by 2 months of daily TRFT (re-balancing) during wakefulness. Not shown are the post-TRFT correlations for VEGF vs. t-tau [r = 0.173; *p* = n.s.] and for VEGF vs. p-tau [r = 0.695; *p* = n.s.]. These non-significant correlations reflect TRFT’s ability to increase low baseline CSF levels of VEGF and do just the opposite for higher baseline CSF levels of VEGF. Graphs reproduced from Ref. [14].

Similar to plasma at baseline, higher levels of baseline VEGF in “CSF” were associated with the increased drainage of t-tau and Aβ1-42 into plasma, as evidenced by 295% and 48% higher levels in plasma for the high VEGF group versus the low VEGF group, respectively [14]. Moreover, there were strong negative correlations in CSF (mLVs) between baseline levels of VEGF and baseline CSF levels of both t-tau and p-tau (Figure 7), consistent with higher levels of VEGF in CSF (mLVs) resulting in the increased clearance of these AD markers from the brain/CSF and into plasma. Sixty days of TRFT during wakefulness eliminated these correlations by clearly increasing CSF VEGF levels in subjects with low baseline VEGF levels and by decreasing CSF levels in subjects with higher baseline VEGF levels.

The soluble AD marker changes (re-balancing) during wakefulness in plasma and CSF after 2 months of TRFT (Figure 6 and Figure 7) appear to be primarily due to modulated (increased) mLV flow and associated brain toxin removal via increases in VEGF, which target mLVs to induce mLV dilation and lymphangiogenesis—both of which modulate mLV flow and toxin removal from the brain into venous blood (Figure 1 and Figure 2) [14]. However, an additional possible way that TRFT modulates “plasma” levels of soluble AD markers does not involve mLVs or VEGF, but rather involves red blood cells (RBCs) being affected by TRFT as they flow through the head and brain underneath the eight RF emitters [33]. Along this line, RBCs concentrate essentially all cytokines, as well as p-Tau, t-Tau, and Aβ, compared to their concentrations in plasma [34]. Since RF waves can induce an increase in membrane fluidity/transport of RBCs [35], TRFT would appear to increase the membrane trafficking of cytokines and AD metabolites, resulting in a flux of a given cytokine/metabolite in or out of RBCs depending on their plasma concentration. Therefore, if a given cytokine/AD metabolite’s concentration in plasma is lower than normal, a net flux of that cytokine across RBC membranes and out of RBCs occurs. It is presently not known which of these two possible mechanisms is more prominent in the re-balancing process.

**Figure 8:**
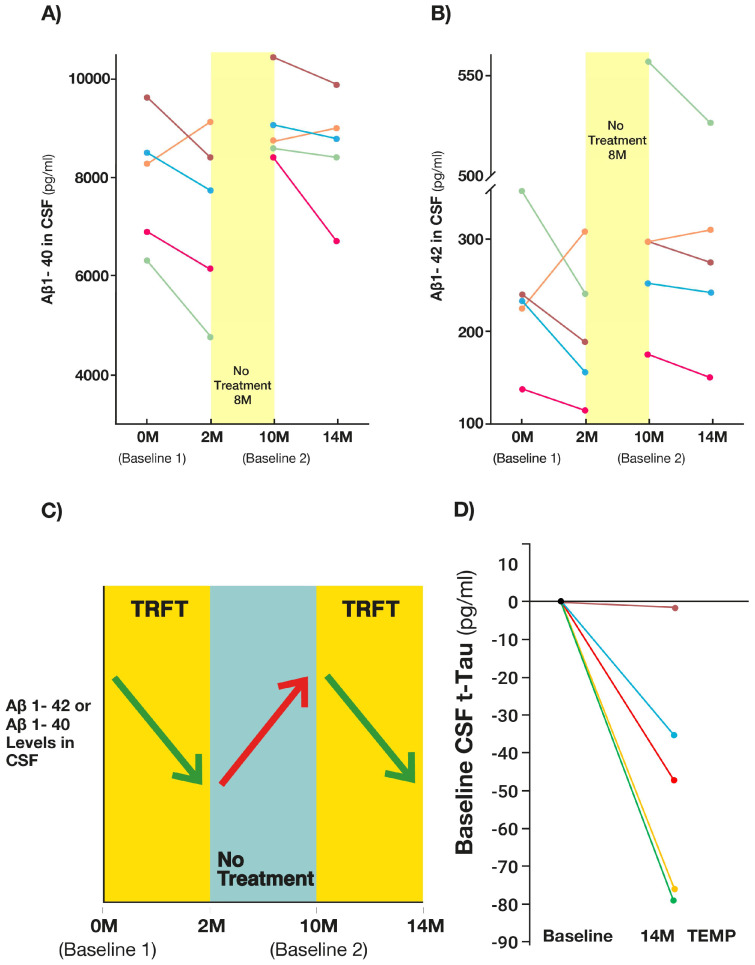
(**A**,**B**) Effects of TRFT over a 14-month period on CSF levels of Aβ1-42 and Aβ1-40. Following an initial 2-month period of daily TRFT, no treatment was given for 8 months, which was followed by a second 4-month period of TRFT. For four of the five AD subjects, decreased levels of both AD markers occurred during periods of treatment and increased levels during the 8-month period of no treatment. The fifth subject (orange) was an outlier in having “plasma” Aβ1-40 and Aβ1-42 levels around 10 times higher than the other subjects. (**C**) A summary of TRFT effects on CSF levels of Aβ1-40 and Aβ1-42. (**D**) Effects of TRFT over a 14-month period on percent change in CSF levels of p-tau217 in five AD subjects. TRFT-induced CSF reductions of 35–79% were seen in four subjects. The fifth AD subject (the outlier in (**A**,**B**)) had plasma baseline p-tau levels 10-fold higher than all others and showed a small decrease in p-tau. All graphs except for (**C**) are reproduced from Ref. [13].

There is also clinical evidence that the brain cleansing ability of TRFT during wakefulness occurs *irrespective of plasma VEGF levels at baseline*. In an extension of the initial 2-month treatment study, AD subjects were given daily TRFT over a 14-month period (with no treatment between 2 and 10 months) and at the same treatment parameters as in the initial 2-month study [13]. At the end of the initial 2 months of TRFT, CSF levels of both Aβ1-40 and Aβ1-42 were significantly reduced in CSF (Figure 8A,B) in four of the five subjects collectively vs. baseline 1 [Aβ1-40 (*p* = 0.012) and Aβ1-42 (*p* = 0.042)]. By the end of the ensuing 8-month period of no treatment in each of these four subjects, CSF levels of both Aβ isoforms rose back up to levels even higher than baseline, while a second 4-month period of TRFT then resulted in CSF levels of both isoforms declining again vs. baseline 2 (Figure 8A,B). Parenthetically, the fifth subject was an outlier in having “plasma” Aβ1-40 and Aβ1-42 levels around 10 times higher than the other subjects, which apparently hampered AD toxin flux out of CSF during TRFT. For the remaining four subjects, their pattern of decrease → increase → decrease in CSF Aβ1-40 and Aβ1-42 (Figure 8C) was also seen for p-tau levels in CSF. The result was a substantial decrease in p-tau levels in CSF compared to baseline at 14 months (Figure 8D). These Aβ1-40, Aβ1-42, and p-tau “CSF” responses to TRFT during a 14-month period are consistent with the enhanced brain drainage/clearance of all three toxins from CSF/mLVs by the end of any given phase of TRFT. The response of “plasma” AD markers to the 14-month period of TRFT [10] was exactly what was seen in the original 2-month TRFT study [14]—namely a re-balancing of AD markers in that TRFT increased low baseline levels and either lowered or did not affect high baseline levels. Examples of this re-balancing of AD markers in plasma after 2 months of TRFT are shown in Figure 6H for t-tau and Aβ1-42.

***Collectively, these results from several clinical TRFT studies summarized in Figure 6, Figure 7 and Figure 8 support an ability of TRFT to substantially increase the removal/clearance of toxins from the brain during wakefulness in aged humans***.

## 5. Brain Toxin Cleansing by TRFT and Wakefulness Are Now Associated

Since a preponderance of AD subjects have low VEGF in their blood [27], TRFT has been proposed to address one of the major dysfunctions of AD and other neurologic conditions [14]—namely, the decrease in toxin cleansing/removal from the brain, thus allowing toxins to build up in the brain, aggregate into toxic oligomers within neurons (in the case of Aβ and tau), and ultimately induce deleterious effect on brain function. It is noteworthy that the 2-month period of TRFT that increased toxin removal from the brain also reversed memory impairment in the same AD patients [30] and re-balanced their immune system in both the brain and blood [31]. In an extended clinical trial, long-term TRFT over a 2½ year period was reported to stop cognitive decline over that period and substantially reduce both brain and blood/body inflammation [13]. It has been proposed that TRFT-induced increases in brain toxin clearance via VEGF actions on mLVs played a significant role in these cognitive and immunologic benefits to AD patients [14].

It is essential to underscore that crucial TRFT effects in relation to “sleep” and “brain cleansing during wakefulness” were inadvertently not recognized or discussed in Arendash et al. [14]. However, it is now evident that the reported TRFT-induced enhancement in the brain clearance of Aβ and tau did not occur during sleep, but rather occurred during wakefulness. The present paper brings to light this important oversight of the previously published work and the resultant implications for brain toxin clearance during wakefulness, particularly in aged individuals. In only now being identified, the apparent beneficial actions of TRFT technology on brain cleansing during wakefulness add credibility to FDA’s “Breakthrough” designation for both the MemoEM device and TRFT technology against AD, in that AD subjects typically obtain less than normal amounts of sleep and have sleep disturbances [6,7,8,9]. It should be noted that the long-term daily administration of TRFT has been shown to have no deleterious side effects in several small AD clinical studies [13,30], as well as in a large body of both human and rodent studies [36].

Together, the published body of TRFT clinical studies in AD subjects [13,14,30] are notable because they are the first to (1) report effects of an intervention on soluble human brain toxin levels (e.g., Aβ, tau), and (2) provide human blood biomarker evidence of increased brain cleansing by a therapeutic intervention. Although these clinical studies involved a small number of subjects, the numerous statistically significant effects observed indicate the robustness of TRFT’s effects on brain toxin cleansing. Moreover, the brain cleansing ability of TRFT was found in humans, providing a level of some confidence that TRFT’s brain cleaning ability will be present for humans in general. It should be noted that all of the aforementioned clinical studies, though supported by private funding provided by NeuroEM Therapeutics, Inc., were performed completely independently by academic institutional collaborators. This involved all blood and CSF sampling/analysis, brain imaging, and complex statistical analyses.

## 6. TRFT: The Only Intervention Shown to Enhance Soluble Toxin Removal from the Human Brain

A review of the animal and human clinical literature indicates that no drug, nutraceutical, or other neuromodulatory device has thus far been shown capable of directly enhancing **endogenous** toxin removal (e.g., Aβ, tau) from the brain into blood during sleep, much less during wakefulness. In this regard, non-pharmacologic interventions have been given primarily to AD mouse models (e.g., transgenic AD mice overexpressing brain Aβ, brain Aβ-infused mice) because of the therapeutic focus for enhancing Aβ clearance from the human brain in AD. Table 1 summarizes such studies of non-pharmaceutical interventions to increase mLV function, and in comparison to related studies involving TRFT in humans. In these mouse interventional studies, no measurement of “soluble” brain toxins such as soluble Aβ, tau, or any other **endogenous** brain toxins/metabolic wastes in plasma or CSF appears to have been performed and to be affected. Rather, the most-utilized endpoint in these mouse interventional studies has been insoluble (deposited) brain Aβ, which is only an indirect measure of toxin removal from the brain. Nonetheless, reductions in brain Aβ deposition have been reported for these various interventions. For example, Borneol is one of the many terpenes found naturally within the cannabis plant, ginger, camphor, and thyme. At 60 min following i.c.v. Borneol treatment into Aβ-infused mice, the drainage of multiple intracerebrally infused macromolecules was increased and two-weeks of oral treatment with Borneol decreased brain Aβ deposition in the same AD model [37]. The Borneol-induced increase in mLV flow occurred through both an increase in mLV diameter and mLV lymphangiogenesis, apparently involving increased VEGF levels. Similarly, in another study, the Chinese herbal medicine Xueshuantong was given orally to AD transgenic (APP/PS1) mice and was found to induce mLV dilation and reduce brain Aβ deposition, though through a non-VEGF mechanism [38].

In addition to TRFT, several other neuromodulatory approaches have been investigated to increase brain toxin clearance (Table 1). In transgenic (5xFAD) mice, fourteen days of repetitive transcranial magnetics stimulation (tMS) improved the drainage of an intracisternally injected tracer from the dura mater and into cervical lymph nodes [39]. This apparent increase in mLV drainage efficiency was accompanied by improved memory in a simple object recognition task and reduced brain Aβ deposition. An additional study of tMS involved a novel form called “continuous theta burst stimulation”, which is generally inhibitory to neuronal activity [40]. In normal mice given this form of tMS acutely, mLVs were dilated via the up-regulation of VEGF in the meninges. The blockade of VEGF receptors eliminated this tMS-induced mLV dilation. Another neuromodulatory approach, transcranial ultrasound (tUS), has also been researched in transgenic (5xFAD) mice. While anesthetized and in a stereotaxic apparatus, these AD mice were given focused tUS sessions once a week for six weeks, with a concurrent vascular infusion of microbubbles to disrupt their blood–brain barrier in the hippocampal region of focused tUS [41]. Although hippocampal Aβ deposition was decreased by the tUS treatment, CSF levels of Aβ1-42 and Y-maze alternation were not affected by tUS.

Multiple studies have involved transcranial/optical photobiomodulation (PBM) in humans [42,43], as well as PBM studies in AD mouse models [26,44]. None have shown an effect on the endogenous brain clearance of either soluble Aβ or soluble tau into blood—neither during sleep nor during the awake state. For example, mice had their mLVs photo-ablated, with an intrahippocampal infusion of Aβ given mid-way between the ensuing seven daily PBN treatments. In indexing the level of mLV flow as the extent of hippocampal Aβ deposition, transcranial PBN increased mLV flow (i.e., PBN resulted in less Aβ deposition when administered during sleep) [44]. However, this increase is well short of returning the mLV flow back to normal levels in these laser-ablated mice. From a practical standpoint, the human cranium’s thickness is a much more substantial barrier to PBM than the mouse cranium, thus requiring “focal” PBN administration for penetration even into superficial human brain regions.

***In view of all the above, TRFT would appear to be the only intervention shown to enhance brain toxin cleansing, as indicated by the actual measurement of soluble brain toxins in CSF and blood. Moreover, TRFT performs this brain toxin cleansing in humans and during wakefulness***.

**Table 1:** A Comparison of Non-Pharmacologic Interventions to Increase Brain Meningeal Lymphatic Function.

Non-Pharmacologic Approach Utilized	Subjects/model and Treatment	Beneficial Brain Effects	Mechanism of Action	Cognitive Effects	References
**Transcranial Radiofrequency Wave Treatment (TRFT; TEMT)**	Humans with AD given over a 2 M–2.5 yr period	↑ Brain clearance of AD markers (Aβ, p-tau, t-tau) (1)	(1)	Reversal and stabilization of cognitive decline	[13,14,30,31,33]
**Borneal**	Mice (Aβ i.c.v.); Borneal via single i.c.v. infusion or orally for 14 days	↑ mLV drainage of i.c.v infused ↓ brain Aβ deposition	(1)	Amelioration of Aβ-induced cognitive deficits	[37]
**Xueshuantong**	Mice (APP/PS1) given i.p. injections for 2–4 wks	↑ mLV diameter (vasodilation) ↓ brain Aβ deposition	(2)	Not evaluated	[38]
**Transcranial Magnetic Stimulation (tMS)**	Mice (5xFAD) given 14 days of tMS	Improved mLV drainage ↓ brain Aβ deposition	(3)	Improved object recognition memory	[39]
	Mice (normal) given acute tMS	Dilation of mLVs	(1)	Not evaluated	[40]
**Transcranial Ultrasound (tUS)**	Mice (5xFAD) given once weekly tUS for 6 wks.	No effect on CSF Aβ levels ↓ brain	(4)	No effect on Y-maze alternation	[41]
**Photobiomodulation (PBM)**	Mice (Aβ infused hippo.) subjected to mLV damage prior to PBM	↓ brain Aβ deposition	(5)	Not evaluated	[44]
Proposed Mechanism of Action:					
Increases VEGF levels in brain and/or blood Increases brain levels of GT-1 (glutamate transport receptor 1) Possible effects on glial cell activity and/or neuroinflammation Decreases gliosis/microglia activation General increase in mLV drainage (no specific mechanism indicated)					

Abbreviations: hippo. = hippocampus; i.c.v. = intracerebroventricularly; M = months.

## 7. Implications of TRFT’s Brain Cleansing for Normal Sleep and Neurologic Disorders

Normal Sleep. If TRFT enhances the brain’s cleansing process during wakefulness (or perhaps even during sleep), it may be possible to sleep less and nonetheless remain healthy longer into advanced age—especially for the many individuals with low blood VEGF levels and/or those who simply do not get the recommended 8–9 h of sleep each day. In essence, TRFT may make removal of brain toxins/metabolic wastes more efficient by not just restricting this process to sleep but extending it into wakefulness. Since the extent to which brain toxin cleaning influences the amount of sleep is currently unknown, it can only be hypothesized that the absolute amount of sleep normally required could be reduced by TRFT. Along that line, there are a few rare genetic mutations that allow humans to sleep only 4–6 h per night for their entire lives while feeling well-rested [45]. What can be said based on the clinical studies discussed in this paper is that TRFT’s actions during wakefulness will likely reduce the amount of sleep required to remove brain toxins/metabolic wastes, especially in aged individuals who generally have impaired mLV flow.

Neurologic Disorders. In the case of individuals with chronic neurodegenerative diseases/conditions characterized by sleep disturbances (e.g., AD), TRFT will likely allow them to get by with less sleep while still attaining the sufficient brain cleansing of toxins. This would be critically important for AD and other neurologic conditions such as Parkinson’s Disease, Lewy Body dementia, Frontotemporal dementia, Amyotrophic Lateral Sclerosis, Corticobasal Disease, Stroke, Traumatic Brain Injury, and Multiple Sclerosis—all of which appear to be caused by the aggregation of soluble protein toxins in the brain (e.g., Aβ, tau, ɑ-synuclein, TDP-43, Huntingtin) into “oligomers” that then induce the pathogenesis of these neurologic diseases/conditions [46,47,48]. Since most of these neurologic conditions are characterized by low brain cleansing (low lymphatic drainage) [3], it is proposed that increasing brain cleansing via TRFT could possibly prevent or lessen the brain accumulation of such soluble toxins to protect against or treat multiple age-related neurologic conditions.

Specifically regarding AD, the primary culprits are soluble Aβ and p-tau oligomeric proteins produced within neurons during aging, that then accumulate in neurons due to an age-related decrease in brain lymphatic drainage/flow. Inside neurons, the result is mitochondrial dysfunction (i.e., low energy production), synaptic loss, and microtubule depolarization—all of which led to neuronal dysfunction/death and the progressive cognitive impairment of AD [48]. With the vast majority of brain cleansing (i.e., Aβ, tau removal) occurring during sleep and very little during wakefulness [1,26], it is thus not surprising that less sleep and sleep disturbances are strongly linked to the development and greater progression of AD [6,7,8,9]. The clinically shown ability of TRFT to enhance brain cleansing during wakefulness [14] appears to have real benefits in AD subjects, as evidence by both a stoppage of their progressive cognitive decline over several years [13] and a reversal of their cognitive impairment [30] over several months of treatment—both as discussed earlier in this paper.

## 8. Implications of TRFT for Insomnia and Sleep Deprivation

Insomnia. A history of insomnia (difficult initiating or maintaining sleep) is linked to decreased brain toxin cleansing and is a significant risk factor for developing AD [6,7,8,9,49,50]. Along this line, a meta-analysis showed that the risk for AD increases nearly 4-fold with pre-existing chronic insomnia [50]. A primary linkage of insomnia to AD involves insomnia-induced increases in CSF levels of Aβ and tau. For example, in a study involving aged individuals with chronic insomnia, those subjects with impaired cognition had increased levels of Aβ1-42, t-tau, and p-tau in their CSF compared to unimpaired chronic insomnias [51]. Also, AD subjects having a medical history of insomnia exhibited greater tau deposition in their brains than AD subjects without such a history [52]. However, “middle-aged” normal subjects with chronic insomnia or high insomnia scores had greater CSF levels only of Aβ1-42, but not of Aβ1-40, t-tau, or p-tau [53,54]—this suggests that Aβ1-42 may be the earliest AD marker to be affected by insomnia.

Results from all of the aforementioned studies are supportive of a decrease in the brain clearance of AD toxins in subjects with chronic insomnia. This, together with the increased production of AD toxins during aging, appears to be a primary mechanism for AD development. Indeed, the resultant increase in “intraneuronal” levels of Aβ and tau precipitates the formation of soluble, toxic aggregates/oligomers of Aβ and tau—the real culprits primary to AD pathogenesis, as previously mentioned [48]. Although TRFT during wakefulness may not decrease the onset and increase the duration of sleep in subjects with chronic insomnia, TRFT’s actions could reduce the amount of sleep required to remove brain toxins/metabolic wastes in chronic insomnia.

Sleep Deprivation. Sleep deprivation can be defined as any forced lack of sleep, such as due to work, pleasure, or involuntarily due to pain. Long-term sleep deprivation during aging can result in the development of various inflammatory [55,56] and neurologic [57] disorders, most notably AD. As is the case for insomnia, sleep deprivation during aging increases the levels of AD markers and metabolic wastes in CSF due to their insufficient removal. For example, healthy middle-aged adults subjected to 36 h of sleep deprivation had substantially increased CSF levels of both t-tau and p-tau during their normal sleep periods [58]. Even a single night of sleep deprivation has significant effects on the brain clearance of the toxins Aβ and tau. For example, Aβ and tau concentrations in normal individuals were measured from blood and CSF collected during sleep-deprived and normal sleep control conditions [59]. During the single night of sleep deprivation, CSF levels of Aβ1-40, Aβ1-42, t-tau, and p-tau were all increased between 35 and 55%, while the plasma levels of these same AD markers were decreased by 5–15% during sleep deprivation. In another study, the normal decline in CSF levels of Aβ1-42 following sleep was eliminated by one night of sleep deprivation in middle-aged men [60]. Amazingly, after a single night of sleep deprivation, healthy humans showed a significant increase in PET-identified (insoluble) Aβ burdens in their brains relative to baseline [61]. As was the case for insomnia, TRFT during wakefulness could reduce the amount of sleep required to remove Aβ and tau from the brain in sleep deprivation.

To summarize, the increased brain Aβ and/or tau levels resulting from insomnia/sleep deprivation (e.g., sleep impairment/deficiency) increase the risk or development of AD [8,49]. Along this line, a meta-analysis of observational studies indicated that around 15% of AD may be attributed to sleep problems [50]. Could a therapeutic intervention such as TRFT decrease the risk or slow development of such neurologic disorders linked to pre-existing sleep impairment/deficiency?

TRFT as a possible treatment for sleep impairment/deficiency? Numerous studies have found that sleep impairment or sleep deficiency can induce a dysfunction of the immune system that can lead to multiple immune-related conditions/diseases [62,63]. Though the interplay between the brain and the immune system is complex, human clinical studies have shown that sleep deficiency induces increases in blood levels of several cytokines (IL-1, IL-6) and an increase in at least peripheral inflammation as measured by blood C-Reactive Protein (CRP) levels [64,65,66]. Surprisingly, however, no human studies have yet determined the effects of sleep deficiency on brain/CSF levels of cytokines (the mediators of immune function) or CRP. This is important, since measuring “brain” immune/inflammatory markers directly via CSF would render the most accurate assessment of sleep deficiency effects on immune function.

How might TRFT actually target the disruption of immune system dysfunction resulting from sleep deficiency? Many brain disorders of aging and aging itself are due, at least in part, to an increase in brain/body inflammation (inflamm-aging), as measured by immune markers [65,66,67]. Inflamm-aging involves the age-related development of an “imbalance” between pro-inflammatory cytokines (overactive) versus anti-inflammatory cytokines (underactive) [68]. Prior to older age, these two immune system components are in balance with one another in young adulthood through middle age, resulting in low inflammation. One of TRFT’s multiple actions in addition to the enhancement of brain toxin clearance is its ability to “re-balance” the immune system’s cytokines in both brain and blood to decrease inflammation [13,31]. For example, daily TRFT to AD subjects for 2 months “rebalances” levels for 11 of 12 cytokines in the blood and/or brain [31]. If a given cytokine’s levels were low in the blood or CSF, TRFT acted to increase those levels and vice versa if the cytokine’s levels were high. In the same subjects, CRP levels measured in both the CSF (brain) and blood at 14 months into TRFT revealed a substantial decline in CRP levels compared to baseline; this occurred with the concurrent stabilization of cognitive function through 14 months and beyond [13].

Thus, TRFT could be a viable treatment for sleep deficiency effects on the immune system by decreasing brain and body inflammation. This would be in addition to TRFT’s other ability of increasing the brain drainage of Aβ and tau [11] to enhance brain toxin cleansing—an ability that should benefit aged individuals with chronic insomnia/sleep deprivation. More broadly, simply improving sleep quality or increasing sleep quantity may be an effective means to reduce brain inflammation and the occurrence, and even treatment, of AD. A spectrum of future TRFT clinical studies involving sleep and brain toxin clearance is now warranted, as proposed in the following section.

## 9. Future Clinical Studies to Further Establish TRFT’s Brain Cleansing Abilities

Although the presently discussed clinical findings related to TRFT’s induction of brain cleansing during wakefulness may be pioneering, they are also preliminary and require follow-up controlled studies in normal subjects for verification. The following future clinical studies are suggested:

(1) To definitively determine if TRFT is inducing enhanced mLV flow, perform direct FLAIR MRI-based measurements of mLV flow in normal humans and AD subjects following acute or several months of daily TRFT compared to their baseline mLV flow.
(2) To determine the 24 h profile of VEGF and brain toxin levels (e.g., Aβ, tau) in the blood following the administration of TRFT during wakefulness compared to placebo, take blood samples through a given 24 h period after acute (a single 1 h treatment) and long-term TRFT (1 h daily treatments for several months).
(3) To determine the extent of brain cleansing provided by TRFT during wakefulness, measure additional brain toxins/metabolic wastes in blood at baseline and following acute or long-term TRFT. Specifically, and in addition to soluble Aβ and tau, measure levels of other brain toxins (e.g., oligomeric Aβ, oligomeric α-synuclein, TDP-43) and metabolic wastes (e.g., lactate).
(4) To determine if TRFT has beneficial effects for insomnia, administer daily TRFT to insomniacs for several months while measuring measures of sleep efficacy and brain toxin cleansing in CSF and blood.
(5) To determine if TRFT’s proposed brain cleansing during wakefulness has benefits on cognition, alertness, and reaction time during sleep deprivation, subject individuals undergoing daily TRFT or controls to sleep deprivation and measure such indices, along with brain toxins.
(6) To determine if TRFT during “sleep” can have beneficial additive effects to sleep on brain toxin cleansing, administer TRFT during sleep for several months and measure plasma and brain toxin levels.

## 10. Knowledge Gaps, Limitations, and Challenges Involving TRFT for Brain Cleaning

As indicated in Section 9, the clinical findings presented in this perspective article related to TRFT’s induction of brain cleansing during wakefulness are preliminary and require follow-up controlled studies for verification. Some of these studies and questions that represent gaps in our knowledge of TRFT’s brain cleansing effects were listed in that section. In addition is the question of how immediate the ability of TRFT to impact mLVs is to thus impact brain toxin clearance during wakefulness? We do know that VEGF levels in plasma are already being modulated after only a single day of TRFT [31], so the modulation of mLVs (and thus brain toxin cleansing) may be rather immediate. Another gap in our knowledge involves TRFT’s modulation of VEGF in the context of brain cancers in that some clinical studies have utilized anti-VEGF agents in an attempt to limit brain tumor vascularization [69], suggesting that increasing VEGF levels may encourage brain cancer growth. However, such agents have been unsuccessful in regressing brain tumors [70] and elevated VEGF levels are in fact associated with benefits against AD cognitive impairment [28]. Indeed, some brain-tumor bearing mouse studies have demonstrated that increasing brain VEGF levels increases mLV flow to increase T cell responsivity at cervical lymph nodes, which then produces a more vigorous immune response against the brain tumor, resulting in tumor regression [71].

Regarding limitations of the currently published TRFT clinical studies [13,14,30,31,33], those clinical studies involved only a limited number of subjects (e.g., 8) and were not controlled. Although it can be argued that having the subject’s own baseline as the control limits treatment variability, the current benchmark for clinical efficacy remains controlled, double-blind clinical trials. Moreover, all of the published TRFT studies involved TRFT treatment to aged subjects with Alzheimer’s Disease, thus raising the question of whether the benefits of TRFT would be seen in normal subjects. As another limitation, the best treatment protocol for TRFT-induced benefits for brain toxin cleansing, Alzheimer’s cognitive impairment, etc. have yet to be established (e.g., Daily or periodic treatment? How long should a treatment period be? Is there a better TRFT frequency or better power level?).

Although TRFT has been clinically administered to humans over a 2½ year period without any safety concerns [13], larger clinical studies monitoring standard safety measures are needed to firmly establish long-term safety. One safety concern that had been raised in the early 2000s was that radiofrequency waves generated by mobile phones increased the risk of brain cancers (i.e., gliomas). However, more recent and very large epidemiologic meta-analyses have clearly shown that is not the case, as summarized by a 2024 report from the World Health Organization (WHO) [72]. As discussed in Baranowski et al. [32], a more realistic safety concern involves the insoluble, deposited Aβ in both cerebrovascular blood vessels and brain parenchyma present in cerebrovascular disease and/or AD. Though unlikely, it is possible that these insoluble aggregates may be dislodged/disaggregated by TRFT, which could result in cerebrovascular blood clots (i.e., stroke) and/or deleterious effects on neuronal function. Only future clinical trials with TRFT can unequivocally establish the long-term safety of TRFT.

Concerning the future challenges for human use of TRFT, the established medical community and pharmaceutical industry both have a bias against neuromodulatory approaches in general, and TRFT in particular—the medical community because they know little or nothing about TRFT and the pharmaceutical industry because an effective TRFT device against aging and age-related diseases would decrease the need for many of their drugs (see next section). Successful clinical trials in the future should go a long way in convincing the medical community of TRFT’s benefits, not the least of which would hopefully be the verification of brain cleansing during wakefulness.

## 11. TRFT’s Brain Cleansing Ability, Along with Its Multiple Other Action, Are Likely to Increase Human Longevity

In a broad sense, “normal aging” itself involves decreased mLV flow/function (i.e., less brain toxin removal), as do a number of neurologic diseases/conditions of aging (e.g., Alzheimer’s Disease, Parkinson’s Disease, Stroke) [15,16,17,18,19]. I have recently proposed that TRFT’s ability to enhance toxin removal from the brain is an important fourth mechanism of TRFT’s action [14] to increase healthy human longevity. The present perspective proposes a further refinement of this fourth TRFT mechanism to now include the rejuvenation of mLV function/flow and the resultant enhancement of brain toxin/metabolic waste removal during “wakefulness”. The other three TRFT mechanisms are as follows: (1) disaggregation of toxic Aβ and tau oligomers in the brain, (2) increased energy production in brain neurons, and (3) re-balancing of both brain and body immune systems to reduce brain/body inflammation [11,30,31,73,74]. The ability of TRFT to increase the brain cleansing/removal of the toxins Aβ and tau is clearly synergistic with mechanism #1 to limit toxin brain levels. As previously discussed, TRFT’s ability to re-balance the immune system to thus reduce inflammation in brain and body [31] could also be beneficial against the immune dysfunction/inflammation characteristic of insufficient sleep [55,56]. Collectively, these four TRFT mechanisms of action not only target a host of age-related diseases and sleep disorders, but target the aging process itself [14,33].

There is already evidence supporting that TRFT may extend the human life span [33]. Relevant to the presently discussed TRFT enhancement of brain toxin removal through VEGF, recent studies have found that functionally increasing VEGF in mice lessened the occurrence of age-associated pathologies (e.g., sarcopenia, osteoporosis, tumors, inflammation), alleviated CNS viral infections, and extended the life span [75,76]. Thus, the novel ability of TRFT to modulate VEGF levels in the blood and brain to impact brain cleansing may be most fortuitous in providing multiple benefits for healthy human aging. It should be noted that the benefits of TRFT need not require continual/daily treatment, since no treatment for weeks or even months does not eliminate the cognitive-enhancing ability of TRFT [13,30]. Thus, periodic treatments after an initial period of daily TRFT would appear to be likely and most practical.

Whether brain cleansing occurs primarily during sleep or can be induced to occur during wakefulness, as is currently being proposed for TRFT, one thing is clear—effective brain cleansing of toxins remains a primary need for a long and healthy lifespan.
